# *Pik3ca* is required for mouse uterine gland development and pregnancy

**DOI:** 10.1371/journal.pone.0191433

**Published:** 2018-01-18

**Authors:** Hye Jin Chang, Hee Sung Shin, Tae Hoon Kim, Jung-Yoon Yoo, Hanna E. Teasley, Jean J. Zhao, Un-Hwan Ha, Jae-Wook Jeong

**Affiliations:** 1 Department of Obstetrics, Gynecology and Reproductive Biology, Michigan State University, Grand Rapids, MI, United States of America; 2 Health Promotion Center, Seoul National University Bundang Hospital, Seongnam, Republic of Korea; 3 Department of Biotechnology and Bioinformatics, Korea University, Sejong, Republic of Korea; 4 Department of Biochemistry and Molecular Biology, Yonsei University College of Medicine, Seoul, Republic of Korea; 5 Department of Biology, Kalamazoo College, Kalamazoo, MI, United States of America; 6 Department of Cancer Biology, Dana-Farber Cancer Institute, Boston, MA, United States of America; Universite du Quebec a Trois-Rivieres, CANADA

## Abstract

The PI3K/AKT signaling pathway plays a critical role in the maintenance of equilibrium between cell survival and apoptosis. The *Pik3ca* gene is mutated in a range of human cancers. It has been found to be oncogenic, and mutations lead to constitutive activation of the PI3K/AKT pathway. The expression patterns of PIK3CA proteins in the uterus of mice during early pregnancy indicate that it may play a role in the regulation of glandular epithelial cells, which is required to support uterine receptivity. To further investigate the role of *Pik3ca* in uterine function, *Pik3ca* was conditionally ablated only in the PGR-positive cells (*Pgr*^*cre/+*^*Pik3ca*^*f/f*^; *Pik3ca*^*d/d*^). A defect of uterine gland development and decidualization led to subfertility observed in *Pik3ca*^*d/d*^ mice. *Pik3ca*^*d/d*^ mice showed significantly decreased uterine weight compared to *Pik3ca*^*f/f*^ mice. Interestingly, a significant decrease of gland numbers were detected in *Pik3ca*^*d/d*^ mice compared to control mice. In addition, we found a decrease of *Foxa2* expression, which is a known uterine gland marker in *Pik3ca*^*d/d*^ mice. Furthermore, the excessive proliferation of endometrial epithelial cells was observed in *Pik3ca*^*d/d*^ mice. Our studies suggest that *Pik3ca* has a critical role in uterine gland development and female fertility.

## Introduction

The endometrial glands are critical for embryo-uterus communication, and their secretions are essential for blastocyst survival, development, and implantation [1–3]. Adenogenesis, the uterine gland formation, is an important developmental process, which determines the receptivity of the adult uterus [3–5]. The organogenesis of mammals is usually established in utero. However, the mouse uterus develops from the Mullerian ducts during the fetal period, and then uterine specific histology is completed in the postnatal period. In mice, adenogenesis occurs after birth. The glands bud from the luminal epithelium. After the budding of the gland, it proceeds to grow into stromal tissue. The folding of luminal epithelium (LE) appears between birth and postnatal day 6 (PD 6), followed by the formation of glandular epithelium (GE) buds [6, 7]. Simultaneously, three layers of mesenchyme are segregated into radially oriented endometrial stroma and inner circular myometrium [8]. By PD 10 in mice, the uterine glands extend from the LE into surrounding endometrial stroma. The histology of the mouse uterus is organized similarly to the adult uterus by PD 15 [9, 10]. Abnormalities in uterine gland development and function result in infertility and miscarriages in human and animal models [11, 12]. The regulation of cell proliferation is involved in postnatal uterus development, as well as the regeneration of cycling endometrium [13]. Uterine luminal and glandular epithelium at the site of implantation should be regenerated after the parturition.

The PI3K/AKT signaling pathway is important for the maintenance of balance between cell survival and apoptosis [14, 15]. Phosphoinositide kinases (PIKs) are lipid kinases, which are categorized into three families: PI3Ks, PIP4Ks and PIP5Ks, according to the phosphorylated site on their carbohydrate. Among those, PI3Ks are further subcategorized into class I, II or III depending on their subunit structure. PI3K is a heterodimeric enzyme consisting of a catalytic subunit (p110) and a regulatory subunit (p85). The *Pik3ca* gene, located on chromosome 3q26.32, codes for the p110a catalytic subunit of PI3K. PI3Kα, which belongs to the class 1A of PI3Ks, has a catalytic domain encoded by *Pik3ca*. Mutations in *Pik3ca* may contribute to the alteration of the PI3K/AKT signaling pathway in several tumors [16]. The mutations are predominantly located in the helical (exon 9) and kinase (exon 20) domains. Several PI3K isoforms have been involved in human disease [16]. *Pik3ca* mutations are frequently detected in endometrial carcinomas [17]. Activation of PI3Kα-regulated signaling led to an increase in dependent or independent AKT pathways in endometrioid endometrial carcinomas [18]. *Pik3ca* mutations were found in 24–36% of endometrial cancer patients, which are associated with poor prognoses such as vascular invasion [19, 20].

Although the effect of *Pik3ca* has been elucidated in tumorigenesis, the role of *Pik3ca* in pregnancy is unknown. In order to evaluate the role of *Pik3ca* in the uterus, we have generated a mouse with *Pik3ca* gene expression that is ablated specifically within the PGR-expressing cells (*Pgr*^*cre/+*^ *Pik3ca*^*flox/flox*^). We demonstrated the expression of *Pik3ca* mRNA during early pregnancy and the effect of *Pik3ca* conditional ablation on implantation and decidualization.

## Materials and methods

### Animals and tissue collection

All mouse experiments were carried out according to the protocol (MSU 11/16-192-00) approved by the Institutional Animal Care and Use Committee of Michigan State University. The surgical procedures have the potential for pain and/or distress post-surgery. Signs of major discomfort include difficulty rising, loss of appetite, and difficulty moving back and forth. To reduce the chances of pain and/or distress in the animals, Ketoprofen was administrated prior to surgery. Post-surgery animals were monitored for several days. Carbon dioxide (CO2) is used for euthanasia.

To assess female reproductive ability, 8-week-old *Pik3ca*^*d/d*^ mice (n = 6) and control female mice (n = 5) were individually mated to wild-type C57BL/3 mice with proven fertility. The numbers of litters and pups were tracked from each female mouse. Copulation was confirmed by vaginal plugs the following morning. The day that a vaginal plug was observed was considered day 0.5 of gestation (GD 0.5). The pregnancy tissues were obtained through the mating of wild-type C57BL/3 mice and *Pik3ca*^*d/d*^, then uterine tissues were flash frozen at the time of dissection or fixed with 4% paraformaldehyde (vol/vol) and paraffin embedded.

For artificial decidualization, control and *Pik3ca*^*d/d*^ female mice (n = 3 per genotype) were ovariectomized under anesthesia at 6 weeks of age. After the duration of at least 2 weeks to eliminate the endogenous ovarian hormone completely, the mice were treated daily with subcutaneous injections (s.c.) of estrogen (E2) (100ng) for 3 days. The mice were treated daily with 1mg of progesterone (P4) s.c. and 6.7 ng of E2 s.c. for 3 days after undergoing 2 days of rest. The left horn of each mouse was mechanically scratched the full length of the anti-mesometrial lumen 6 hours after the third P4 and E2 injection, while the right horn was left unstimulated as an adjusted control. A 25 gauge sterile needle was inserted into the uterine lumen through the uterine wall near the tubal utero junction. The end of the needle was brushed against the antimesometrial uterine wall to traumatize the uterus of one uterine horn. Ketoprofen was administrated before the skin incision is made. Mice were anaesthetized with isoflurane gas by inhalation. To induce the maximal decidual reaction, the mice continued daily treatment with s.c. injections of 1 mg P4 and 6.7ng E2 for 5 days after the mechanical stimulation. The wet weights of the stimulated and control uterine horns of each mouse were measured. Uterine tissues were then collected from both horns and fixed in 4% paraformaldehyde (vol/vol) and paraffin embedded. To investigate the function of *Pik3ca* on uterine gland development, uteri of *Pik3ca*^*d/d*^ mice were collected for histologic evaluation at 20 days and 8 weeks of age.

### Immunohistochemistry

Uterine sections were incubated with 10% normal goat serum in PBS for 2 hours. Sections were exposed to primary antibody anti-PIK3CA (CS-4252, Cell Signaling), anti-FOXA2 (WRAB-FOXA2, Seven Hills Bioreagents), anti-Ki67 (BD550609, BD Bioscience) in blocking solution overnight at 4°C. After secondary antibody (Vector Laboratories, Burlingame, CA) incubation, sections were treated with the horseradish peroxidase-conjugated streptavidin substrate. We detected positive signaling with the Vectastain Elite DAB kit (Vector Laboratories). Proliferation and the number of uterine glands was quantified by counting the number of glands and proliferation-positive cells in the uterine sections.

### RNA isolation and quantitative real-time PCR

Total RNA was isolated from the uteri using a Qiagen RNeasy total RNA isolation kit (Qiagen, Valencia, CA, USA). The expression of *Pik3ca*, *Foxa2*, and *Spink3* was quantified by real-time PCR TaqMan (*Foxa2*) and SYBR green (*Pik3ca* and *Spink3*) analysis using an Applied Biosystems StepOnePlus system. It was based upon the manufacturer’s instructions (Applied Biosystem, Foster City, CA, USA). Template cDNA was generated from 1 μg of total RNA using random hexamers and MMLV Reverse Transcriptase (Invitrogen Corp.). Standard curves were produced by serial dilution of a prepared sample of total RNA isolated from the mouse uteri. The results of real-time RT-PCR were normalized against pre-validated probes, primers, and 18S RNA using an ABI Universal Master mix reagent.

### Western blot analysis

Isolation of uterine tissue and western blot were perfomed as described previously [21]. Briefly, total 20μg protein was loaded onto gels and transferred to membrane. The membrane was incubated with 0.5% (w/v) casein (Sigma Aldrich) for 3 hours and then treated with anti-FOXA2 (WRAB-FOXA2, Seven Hills Bioreagents). Total Actin (SC1616, Santa Cruz Biotechnology, Inc.) levels were used for loading controls.

### Statistical analysis

For data that contained more than two groups, a parametric Tukey-Kramer one-way ANOVA was used to test the null hypothesis of group differences, followed by a Wilcoxon test. For data from two groups, an unpaired t-test was used. All data is presented as mean ± SEM. *P* < 0.05 was considered statistically significant. All statistical analysis was performed by Instat package from GraphPad (San Diego, CA, USA).

## Results

### The expression of *Pik3ca* during early pregnancy

**Fig 1:**
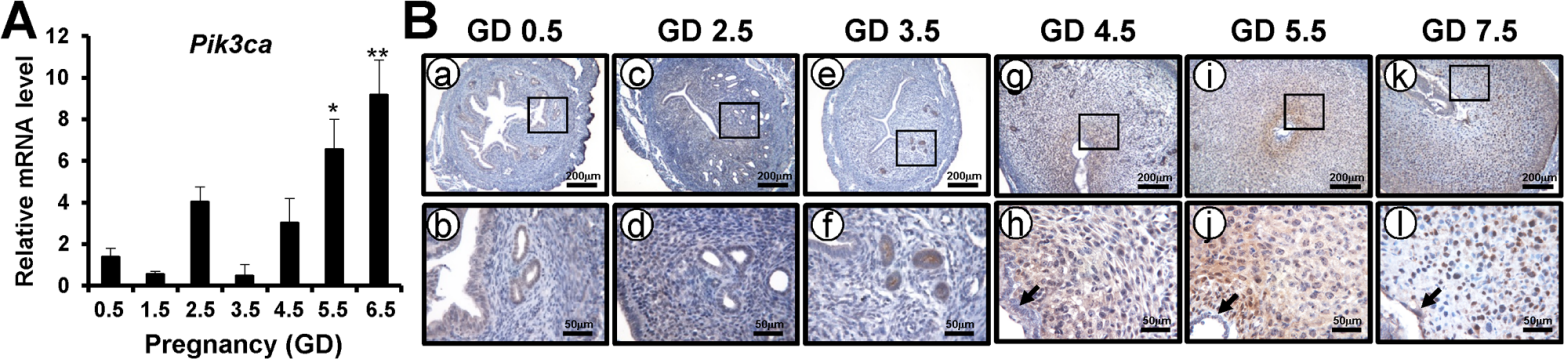
The expression of *Pik3ca* mRNA and protein from GD 0.5 to GD 7.5. (A) The mRNA expression of *Pik3ca* was investigated in the uteri of C57BL/6 mice during early pregnancy. *Pik3ca* expression was significantly increased at GD 5.5 and GD 6.5. (A) The localization pattern of PIK3CA proteins was investigated through immunohistochemistry in uteri of C57BL/6 mice on GD 0.5 (a and b), GD 2.5 (c and d), GD 3.5 (e and f), GD 4.5 (g and h) GD 5.5 (i and j) and GD 7.5 (k and l). An arrow indicates an embryo. The results represent the mean ± SEM. **, p < 0.01; *, p < 0.05.

The expression of *Pik3ca* was examined in the uterus from gestational day (GD) 0.5 to GD 6.5 by real-time RT-PCR (Fig 1A). There was a peak in *Pik3ca* expression at GD 2.5 and then it sharply decreases at GD 3.5. The expression of *Pik3ca* gradually increased from GD 3.5 until GD 6.5. The expression of *Pik3ca* was significantly increased at the time of implantation and continues to increase as pregnancy progresses indicating an important role for *Pik3ca* in pregnancy. Next, we examined the spatial expression profile of PIK3CA protein during early pregnancy using immunohistochemistry (Fig 1B). The expression of PIK3CA proteins were weakly detected at the luminal epithelium, glandular epithelium, and stromal cells but not myometrium at GD 0.5 and GD 2.5. Interestingly, PIK3CA proteins were strongly expressed at the glandular epithelium of GD 3.5. After embryo implantation, the expression of PIK3CA was remarkably strong in decidual cells.

### Ablation of *Pik3ca* in mouse uterus

**Fig 2:**
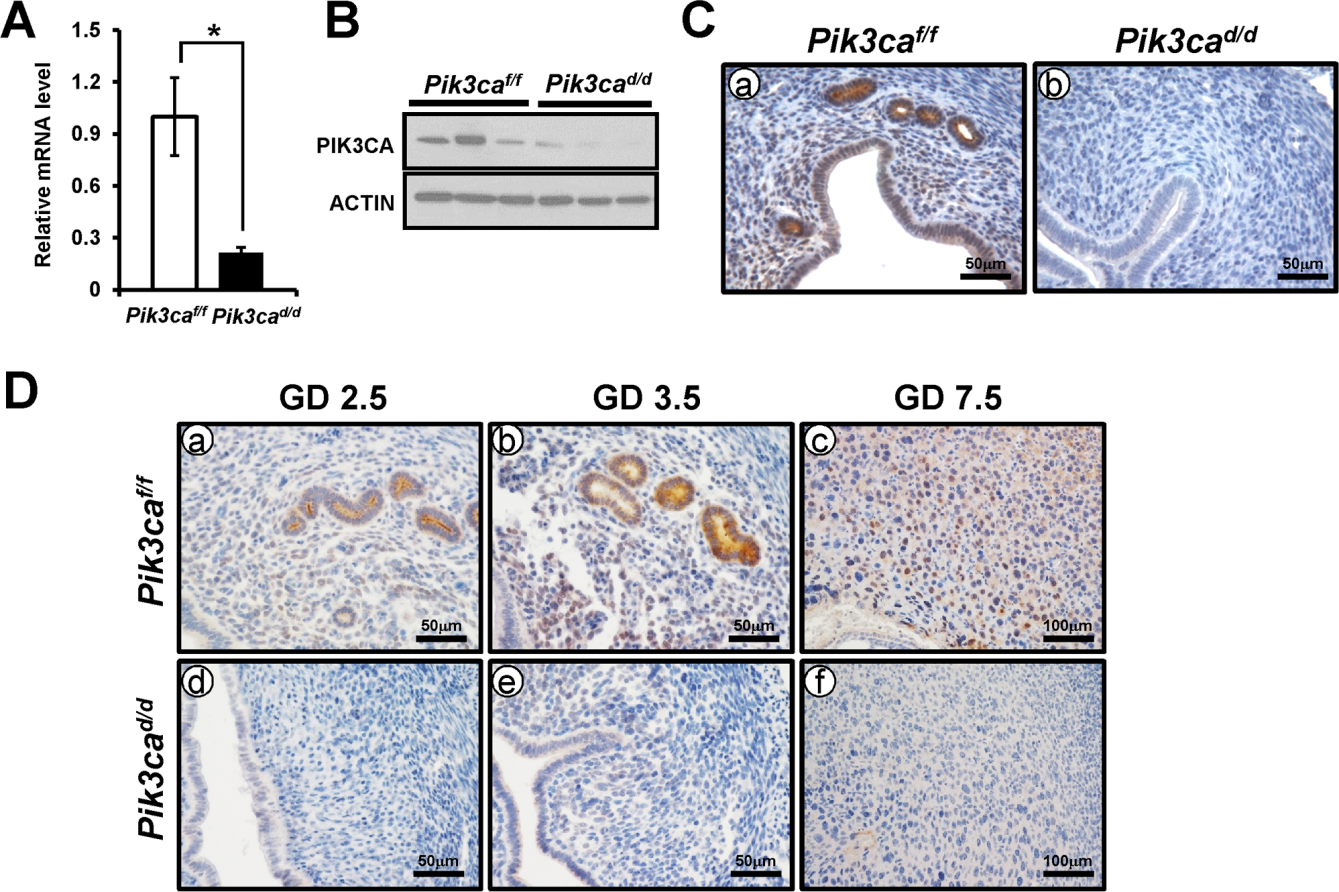
The generation of mouse with uterine specific *Pik3ca* ablation. (A) The expression of *Pik3ca* mRNA was evaluated in uteri by real time PCR. The data represents the mean ± SEM. *, p < 0.05. PIK3CA protein expression was assessed by Western blot (B) and immunohistochemistry (C). Total RNA and protein were prepared from whole uteri. (D) The efficiency of PIK3CA loss was evaluated in uterus of *Pik3ca*^*d/d*^ mice at GD 2.5 (a and d), GD 3.5 (b and e), and GD 7.5 (c and f).

To investigate the function of *Pik3ca* in the uterus, we generated a mouse model with the uterine *Pik3ca* ablation in the PGR-expressing cells (*Pgr*^*cre/+*^ *Pik3ca*^*f/f*^; *Pik3ca*^*d/d*^) [22, 23]. Ablation of *Pik3ca* was confirmed in the uteri from 8 weeks old mutant *Pik3ca*^*d/d*^ mice by real time PCR, Western blot, and immunohistochemical analysis (Fig 2). *Pik3ca* mRNAs were significantly decreased in uteri of *Pik3ca*^*d/d*^ mice compared to *Pik3ca*^*f/f*^ mice. To assess the efficiency of ablation, the levels of PIK3CA proteins were examined during early pregnancy. Expression of PIK3CA proteins was not observed in the uterus of *Pik3ca*^*d/d*^ mice at GD 2.5, GD 3.5, and GD 7.5 (Fig 2D). These results confirm our successful ablation of *Pik3ca* in the uterus of *Pik3ca* ^*d/d*^ mice.

### *Pik3ca*^*d/d*^ mice are subfertile due to impaired implantation

Female fertility was assessed by mating *Pik3ca*^*d/d*^ (n = 6) and control females (n = 5) with wild type males continuously for 6 months and follow up of the quantification of litters and pups from each female mouse. *Pik3ca*^*d/d*^ mice were subfertile with only 1.33 ± 0.49 litters/mouse compared with 4.20 ± 0.58 litters/mouse from control mice (Table 1). Control mice had an average 7.43± 0.43 pups/litter, whereas *Pik3ca*^*d/d*^ mice had 3.75±0.80 pups/litter. *Pik3ca*^*d/d*^ females produced significantly fewer pups per mouse (p<0.001), fewer litters per mouse (p<0.01), and fewer pups per litter (p<0.01) than the control females.

**Table 1:** *Pik3ca*^*d/d*^ mice were subfertile.

Genotype	Number of Mice Tested	Number of Litters	Number of Pups	Average of Pups/Litter	Average Number of Litter/mouse
*Pik3ca*^*f/f*^	5	21	156	7.43 ± 0.43	4.20 ± 0.58
*Pik3ca*^*d/d*^	6	8	30	3.75 ± 0.80	1.33 ± 0.49

To test for a possible ovarian defect, blastocysts generated by natural mating were collected at GD 3.5 by uterine flushing with M2 medium, and counted. The number of blastocysts from uteri of *Pik3ca*^*f/f*^ and *Pik3ca*^*d/d*^ mice was not different. Also, histological analysis of the *Pik3ca*^*d/d*^ ovary did not show any alterations in ovarian morphology (S1 Fig). These results show that ovarian morphology and functioning were not affected in the *Pik3ca*^*d/d*^ mice, suggesting that the fertility defect is primarily due to a uterine defect.

**Fig 3:**
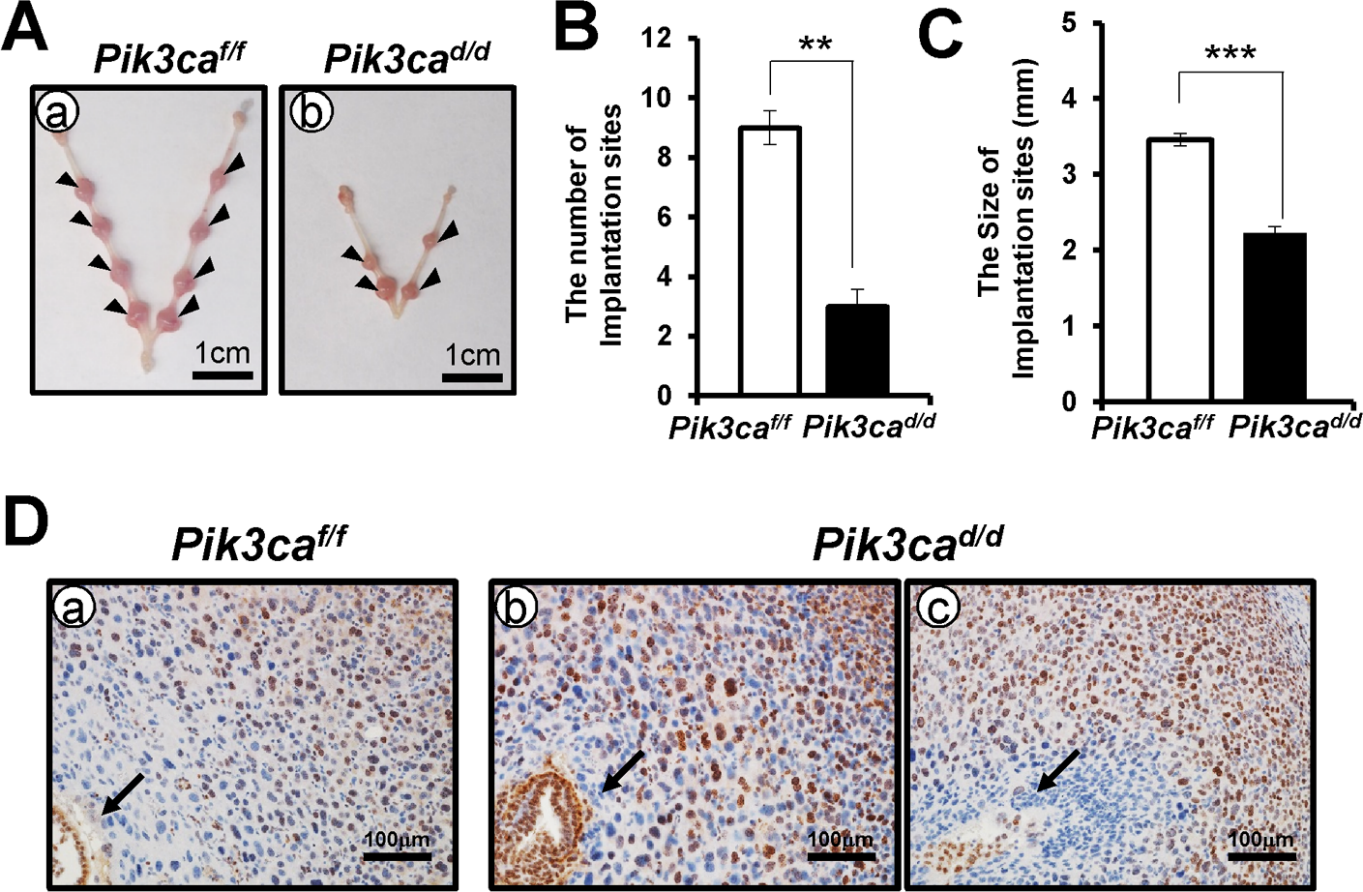
*Pik3ca*^*d/d*^ mice have impaired implantation. (A) Gross anatomy of implantation sites in both the control (a) and *Pik3ca*^*d/d*^ uterus (b) at GD 7.5. (B) The number of implantation sites were recorded in the uteri. (C) The size of implantation sites were measured. (D) The proliferative cells were assessed in control and *Pik3ca*^*d/d*^ uterus at GD 7.5 by immunohistochemical staining using anti-Ki67 antibody. Arrow indicates the blastocyst. The data represents the mean ± SEM. ***, p < 0.001; **, p < 0.01.

To investigate whether *Pik3ca* plays an essential role in implantation, female control and *Pik3ca*^*d/d*^ mice were mated with wild-type C57BL/3 mice and then the mice were examined at the morning of GD 7.5 to evaluate embryo implantation. A significant decrease in the number of implantation sites was observed in the *Pik3ca*^*d/d*^ mice (3.0 ± 0.6) compared with the control (9.0 ± 0.6) (Fig 3A and 3B). These results revealed that defect of implantation is one of the causes of the subfertility observed in *Pik3ca*^*d/d*^ mice

In addition, the size of implantation sites was significantly decreased in *Pik3ca*^*d/d*^ mice (control vs mutant; 3.4 ± 0.2 vs 2.2 ± 0.1). (Fig 3C). However, the proliferation of stromal and decidual cells was not different in *Pik3ca*^*d/d*^ mice (57.11 ± 1.40%) compared with control mice (56.72 ± 2.51%) at GD 7.5. The average of implantation sites (3.50 ± 0.50) and the average of pups per litter in *Pik3ca*^*d/d*^ mice (3.75 ± 0.80) were not different. These results suggest that *Pik3ca*^*d/d*^ mice do not have any defect after implantation.

### Aberrant proliferation of epithelial cells in *Pik3ca*^*d/d*^ mice at the pre-implantation stage

**Fig 4:**
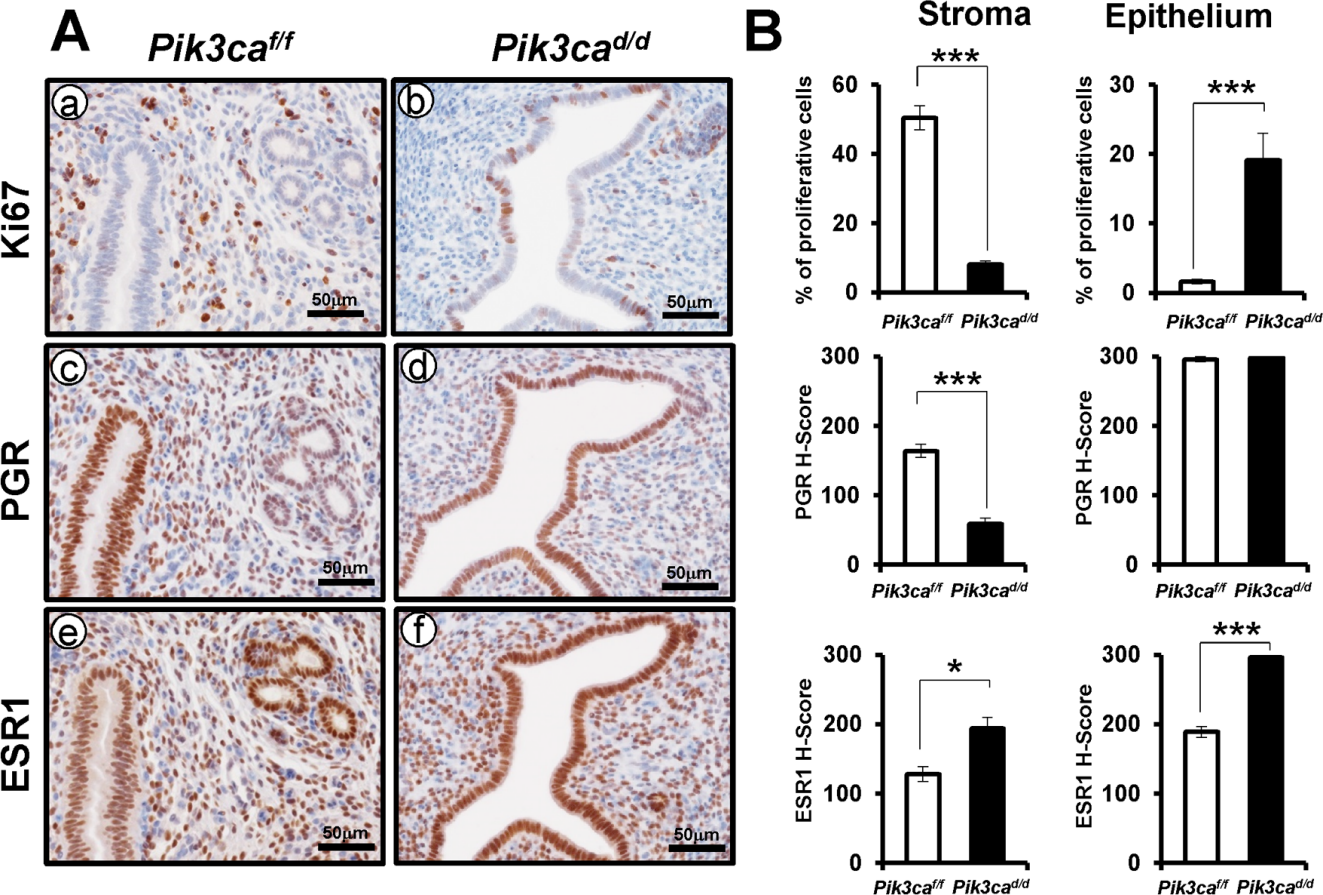
Proliferation of endometrial cells was dysregulated in the uteri of *Pik3ca*^*d/d*^ mice at the pre-implantation stage. (A) Immunohistochemical analysis of Ki67 (a and b), PGR (c and d), and ESR1 (e and f) proteins at GD 3.5 in endometrium of control (a, c, and e) and *Pik3ca*^*d/d*^ (b, d, and f) mice. (B) Quantification of Ki67 positive cells and semi-quantitative analysis of PRG and ESR1 in uterine epithelial and stromal cells of control and *Pik3ca*^*d/d*^ mice. The results represent the mean ± SEM. ***, p < 0.001; *, p < 0.05.

In the rodent uterus, E2 induces the proliferation of luminal and glandular epithelial cells at GD 1.5–2.5 [24]. Such cell proliferation is redirected from the epithelial to the stromal cell by P4 stimuli after GD 3.5 [25]. Stromal cells proliferate and differentiate to specialized maternal tissue referred to as the decidua that should be necessary to maintain pregnancy. To determine whether the proliferation of endometrial cells is dysregulated in the pre-implantation period by ablation of *Pik3ca*, we examined the change in the spatial distribution of proliferative marker Ki-67 in the uteri of *Pik3ca*^*d/d*^ mice at GD 3.5 using immunohistochemical staining (Fig 4A). The proliferation is significantly increased in the endometrial epithelial cells of *Pik3ca*^*d/d*^ mice compared with control mice. Furthermore, proliferation of endometrial stromal cells showed a significant decrease in *Pik3ca*^*d/d*^ mice compared to control (Fig 4B). This suggests that *Pik3ca* has an essential role in regulating proliferation of endometrial epithelial and stromal cells for successful implantation.

Somatic mutations of the PIK3CA gene have been shown to activate AKT and induce oncogenic transformation in vitro and in vivo [26–28]. Therefore, we examined the levels of pAKT and total AKT in *Pik3ca*^*f/f*^ and *Pik3ca*^*d/d*^ mice using immunohistochemistry. However, the levels of pAKT and total AKT were not changed in *Pik3ca*^*d/d*^ mice compared to *Pik3ca*^*f/f*^ mice at GD 3.5 (S2 Fig).

Since an increase of epithelial proliferation is observed in *Pik3ca*^*d/d*^ mice, we further investigated whether *Pik3ca* ablation altered the expression of PGR and ESR1. Interestingly, stromal PGR expression was significantly reduced in *Pik3ca*^*d/d*^ mice compared to *Pik3ca*^*f/f*^ mice. We also observed an increase of ESR1 protein expression in the stroma and epithelium of the *Pik3ca*^*d/d*^ mice compared to the *Pik3ca*^*f/f*^ mice. These results suggest that *Pik3ca* loss causes dysregulation of PGR and ESR1 expression in the uterus.

### *Pik3ca*^*d/d*^ mice exhibited a defect of decidualization

**Fig 5:**
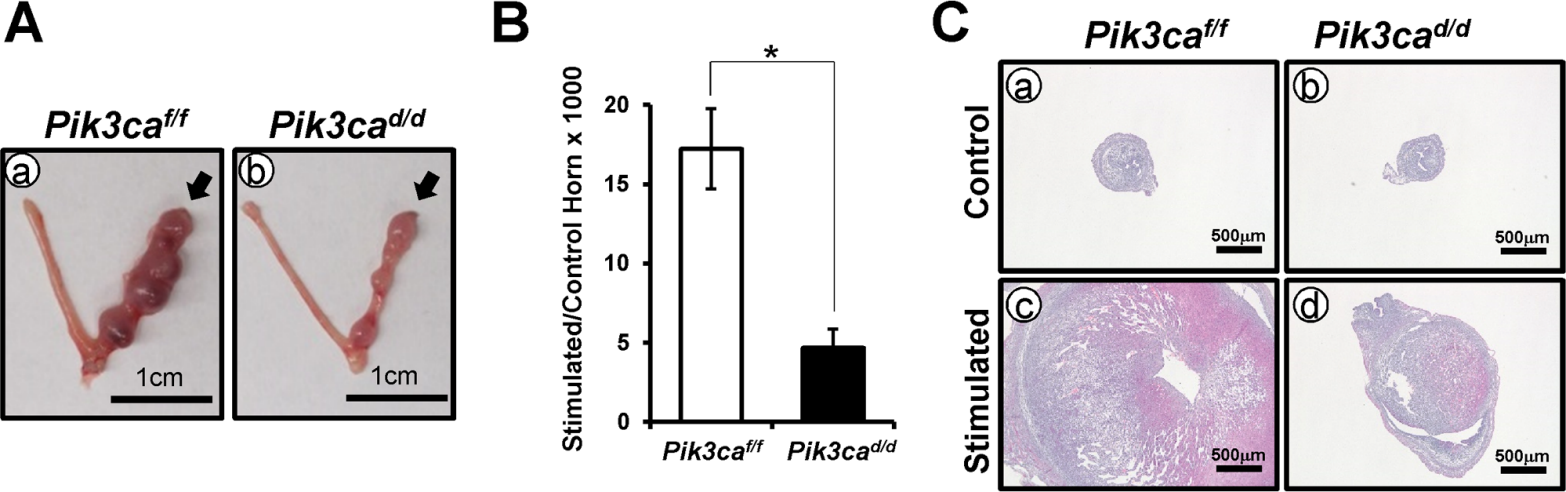
*Pik3ca*^*d/d*^ mice exhibited a defect of decidualization. (A) Gross anatomy of artificially induced decidualized uteri of control (a) and *Pik3ca*^*d/d*^ (b) mice. Arrow head indicates a stimulated horn. (B) There was a significant decrease in stimulated and unstimulated (control) horn weight ratio in *Pik3ca*^*d/d*^ mice as compared with control. The results represent the mean ± SEM. *, p < 0.05. (C) Histology of control (a and b) and stimulated (c and d) horn in control (a and c) and *Pik3ca*^*d/d*^ mice (b and d) at day 5.

Implantation is an important step in the establishment of a pregnancy, where the blastocyst attaches to the endometrial epithelium and invades to the differentiated endometrial stromal cells; the decidual cell. To investigate whether the ablation of *Pik3ca* affects decidualization, control and *Pik3ca*^*d/d*^ mice underwent experimentally-induced decidualization after hormone treatment. Ovariectomized *Pik3ca*^*d/d*^ (n = 3) and control (n = 3) mice were treated with E2 and P4, and the uteri were stimulated by scratch to mimic embryo attachment and to induce decidualization [21]. The gross anatomy and histology of the *Pik3ca*^*d/d*^ uteri 5 days after having artificially induced decidualization revealed that the ratio of stimulated to unstimulated horn weight in mutant uteri was significantly decreased compared to control mice (Fig 5). The results demonstrate that *Pik3ca*^*d/d*^ mice exhibit a defect of decidualization.

### *Pik3ca*^*d/d*^ mice exhibit a defect of uterine gland formation

**Fig 6:**
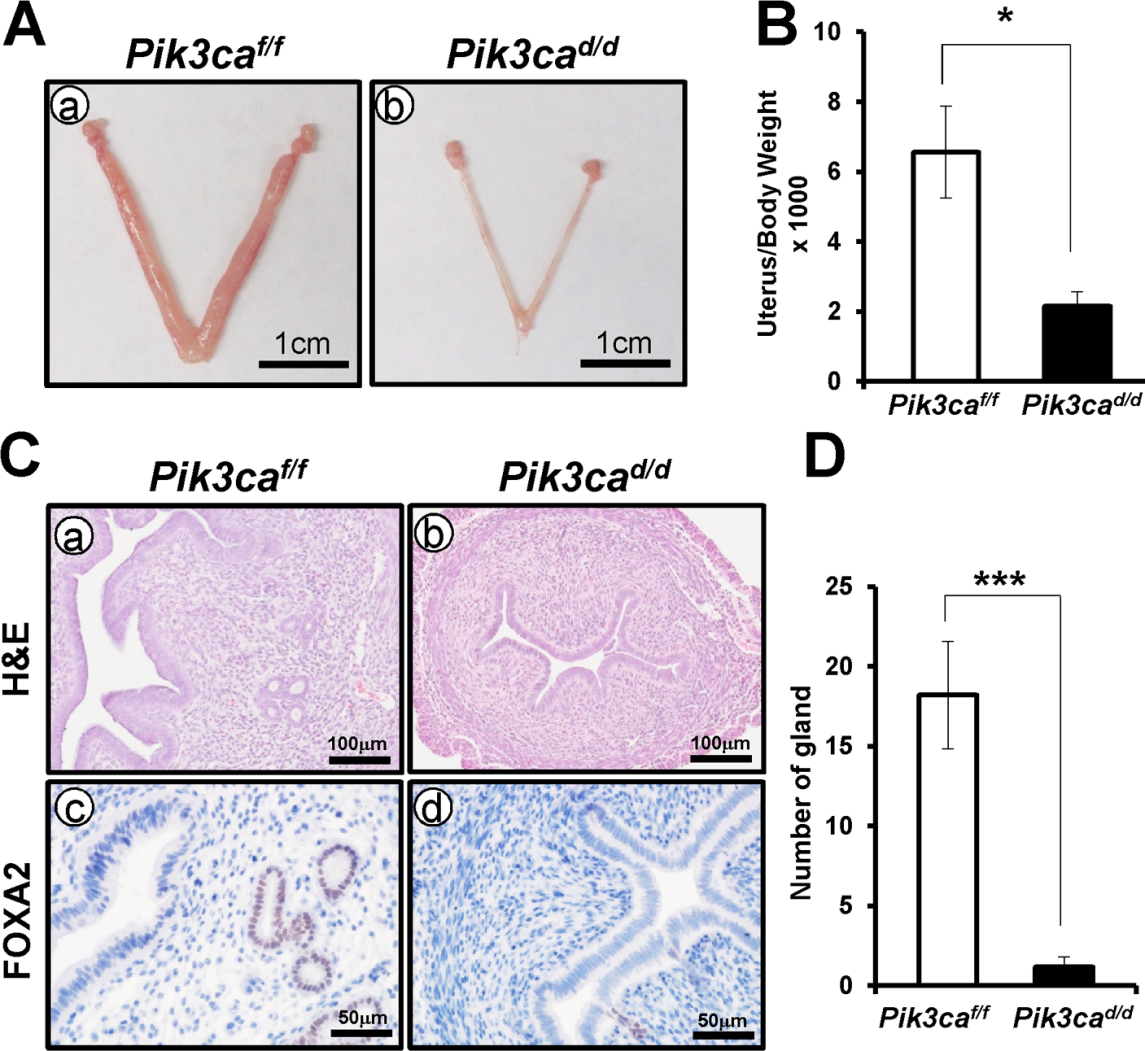
The defect of gland formation in *Pik3ca*^*d/d*^ mice. (A) Gross anatomy of 8-week-old control (a) and *Pik3ca*^*d/d*^ (b) mice. (B) There was a significant decrease in the ratio of uterine weight in *Pik3ca*^*d/d*^ mice. (C) Histology of the uterus of control (a) and *Pik3ca*^*d/d*^ (b) mice. The expression of FOXA2 in the uterus of control (c) and *Pik3ca*^*d/d*^ (d) mouse. (D) Quantification of FOXA2 positive endometrial glands in mouse uteri. The results represent the mean ± SEM. *, p < 0.05; ***, p < 0.001.

To investigate the cause of subfertility in *Pik3ca*^*d/d*^ mice, we next examined the uterine development in *Pik3ca*^*d/d*^ mice. Gross examination revealed that there was a significant decrease in the uterine size and weight in *Pik3ca*^*d/d*^ mice when compared to the control at 8 weeks of age (Fig 6A and 6B). Interestingly, we found a defect of uterine gland development in *Pik3ca*^*d/d*^ mice by histological analysis (Fig 6C). To validate uterine gland formation, we examined the expression of FOXA2, a uterine gland marker [29], by immunohistochemical analysis (Fig 6C). There was a significant reduction in the number of uterine glands in *Pik3ca*^*d/d*^ mice compared to control mice (Fig 6D).

**Fig 7:**
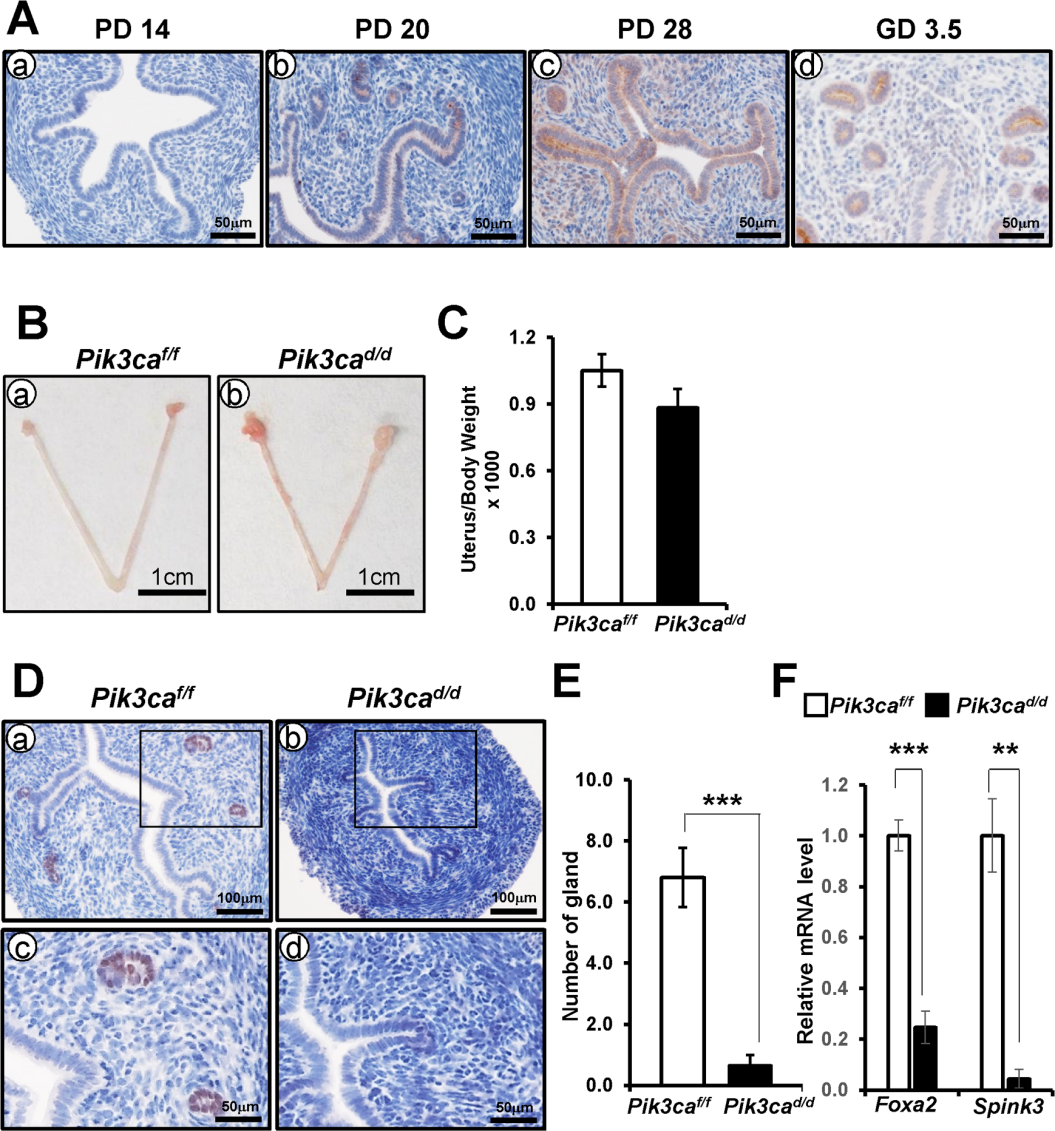
A defect of uterine gland development in *Pik3ca*^*d/d*^ mice at 20 days of age. (A) The expression of PIK3CA was examined during uterine development. Immunohistochemical staining of PIK3CA in the uterus of PD 14 (a), PD 20 (b), and PD 28 (c). The uterus of GD 3.5 was used a positive control (d). (B) Gross anatomy of control (a) and *Pik3ca*^*d/d*^ (b) mice at PD 20. (C) The weight of uteri was not different between control and *Pik3ca*^*d/d*^ mice. (D) Immunohistochemical staining of FOXA2 in control (a and c) and *Pik3ca*^*d/d*^ (b and d) mice. (E) Quantification of FOXA2 positive endometrial glands in uteri of control and *Pik3ca*^*d/d*^ mice at PD 20. (F) The expression of *Foxa2* and *Spink3* in the uteri of control and *Pik3ca*^*d/d*^ mice. The results represent the mean ± SEM. ***, p < 0.001; **, p < 0.01.

To better understand the role of PIK3CA in uterine gland, we examined the expression of PIK3CA during uterine gland development. As illustrated in Fig 7A, immunohistochemistry analysis revealed that PIK3CA positive cells were not observed on PD 14, but were observed by PD 21, only in some luminal and glandular epithelial cells. The expression of PIK3CA was increased and extended to all luminal and glandular epithelial cells by PD 28. To investigate the defect of glandular development in *Pik3ca*^*d/d*^ mice, we examined the uterine histology of peripubertal mice (PD 20). Murine uterine glands extend from the luminal epithelium into surrounding endometrial stroma by PD 10, and the histoarchitecture of the mouse uterus resembles that of the adult by PD15 [9, 10]. Therefore, PD 20 mice could have just completed uterine morphology. There was no difference in the size and weight of the uteri between the mice at PD 20 (Fig 7B and 7C). However, histological analysis and immunohistochemical analysis of FOXA2 showed that the number of endometrial glands was significantly decreased in *Pik3ca*^*d/d*^ mice compared to control mice at PD 20 (Fig 7D and 7E). Furthermore, glandular epithelium expressed genes *Foxa2* and *Spink3* were significantly decreased in uteri of *Pik3ca*^*d/d*^ mice at PD 20 (Fig 7F). These results suggest that *Pik3ca* plays an important role in uterine glandular development.

## Discussion

This study has revealed that *Pik3ca* has an important role in early pregnancy and uterine gland development. The expression of *Pik3ca* is significantly increased at the time of implantation and continues to increase as pregnancy progresses indicating a critical role for *Pik3ca* in pregnancy. PIK3CA protein had been strongly detected during decidualization. *Pik3ca*^*d/d*^ mice were subfertile due to impaired implantation and decidualization. *Pik3ca*^*d/d*^ mice showed a decrease of implantation sites and dysregulated proliferation at the time of pre-implantation, as well as impaired decidualization in *Pik3ca*^*d/d*^ mice.

Mutations of *Pik3ca* are frequently detected in endometrial cancer. When PI3Kα-mediated signaling is inappropriately activated, AKT-dependent or AKT-independent signaling is increased. The PI3K/AKT signaling pathway is a major regulator in maintaining the equilibrium between cell survival and apoptosis. PI3K, along with downstream effectors such as AKT1 and mammalian target of rapamycin (mTOR), have been regarded as a possible target for cancer therapy [16]. However, PI3K-AKT-mTOR pathway signaling works in many normal tissues as well as malignant cells. PI3K is common to the signaling pathways of a number of peripheral metabolic mediators, including insulin and leptin, which regulate both metabolism and reproduction [30]. Male mice of *Pik3ca* knockout in adipose tissue displayed delayed onset of puberty accompanied by a reduction in plasma LH levels and testicular weight [31]. We could not observed dysregulation of AKT and pAKT in *Pik3ca*^*d/d*^ mice. However, the lack of altered expression of total AKT and phospho-AKT at the preimplantation of *Pik3ca*^*d/d*^ mice (S2 Fig) does not rule out the possibility that *Pik3ca* regulates adenogenesis through PI3K/AKT signaling during the neonatal period, when glands are initially formed. Therefore, Future studies are needed to determine the potential role of complex interactions between *Pik3ca* and PI3K/AKT signaling to either initiate or maintain glandular identity.

The uterine glands are known to be derived from and remain connected to the luminal epithelium [12]. Prolactin and estradiol-17 beta may be involved in endometrial adenogenesis of neonatal ungulates [9, 12]. This could play a role in the regulation of human endometrial gland regeneration after menstruation. On the other hand, P4 exposure suppresses endometrial adenogenesis in the neonatal uterus by modulating expression of gland development related genes and cell proliferation [32]. Perinatal uterine development is a critical period for future reproductive health, and exposure to endocrine disruptors can have long term adverse consequences [33]. Mouse models in which *Wnt5a*, *Wnt7a*, and β-catenin (*Ctnnb1*) were ablated all displayed a lack of uterine glands [34–37]. PIK3CA mutations were associated with over-expression of several genes involved in the Wnt signaling pathway [38]. The reciprocal interaction between *Pik3ca* and Wnt signaling has been noted also in that *Ctnnb1*, a downstream effector of the Wnt pathway, can be promote by oncogenic PIK3CA [39]. Future studies are needed to determine the potential role of complex interactions between Wnt/β-Catenin and PIK3CA signaling to either initiate or maintain glandular identity.

We found that peripubertal uteri of *Pik3ca*^*d/d*^ mice showed decreased *Foxa2* and *Spink3* mRNA expression. *Foxa2* is known as a critical gene in the formation of glands in the uterus. *Foxa1* and *Foxa2* are necessary for normal development of endoderm-derived organs [40–43]. In reproductive organs, the ovary does not express *Foxa1* and *Foxa2*, and only glandular epithelium of the uterus expresses *Foxa2* [44]. *Foxa2* induces epithelial specific gland bud formation during postnatal uterine development. The uteri of adult FOXA2-deleted mice were morphologically normal and contained glands, whereas the uteri of neonatal FOXA2-deleted mice were completely aglandular [45, 46]. *Foxa2* transcriptional factors are able to bind chromatin and induce chromatin decondensation, which facilitates the binding of other transcription factors, such as glucocorticoid, androgen, and estrogen receptor 1 [47].

Serine peptidase inhibitor, Kazal type 3 (*Spink3*) is a trypsin inhibitor, and also a growth factor that has a similar structure to epidermal growth factor (EGF), which could bind with epidermal growth factor receptor (EGFR) to promote cell proliferation [48]. *Spink3* was likely to promote hepatocytes proliferation in liver regeneration through P38, PKC, JAK-STAT and AKT [49]. *Spink3*, which is derived from the uterine glands, is involved in endometrial decidualization and implantation [50]. In this study, decreased expression of *Foxa2* and *Spink3* mRNA in peripubertal period of *Pik3ca*^*d/d*^ mice might be involved in postnatal uterine gland formation.

Ovarian hormones stimulate uterine cell proliferation by various mechanisms, such as induction of growth factors, and paracrine signaling, and by direct regulation of cell cycle genes [51]. Tissue-specific knockout of the *Pgr* in the epithelial cells of mouse uteri resulted in the continued proliferation of epithelial cells in the absence of P4 action [52]. In addition, the proliferative switch from epithelial cell to stroma also occurred normally at GD 3.5 in mice. Stromal cell proliferation and differentiation are prerequisites to a receptive uterus for embryo implantation. Our results showed that the proliferation of stromal cells were significantly decreased in *Pik3ca*^*d/d*^ mice at GD 3.5 compared with control mice. This indicates that *Pik3ca* might have an important bearing on the regulation of stromal cell proliferation. In addition, *Pik3ca* mutant mice had the uterine phenotype of decreased endometrial glands. Our study supports that the disruption of uterine development during critical organizational periods can alter the functional capacity and embryotrophic potential of the adult uterus.

In summary, we have generated a mouse model with conditional ablation of *Pik3ca* in the uterus. Uterine-specific knockout of *Pik3ca* demonstrates a defect in uterine gland formation. The mutant mice were subfertile with impaired implantation and decidualization. This study supports that *Pik3ca* is an important gene for early pregnancy, and improves the understanding of possible effects of anticancer therapy targeting *Pik3ca* signaling on female reproduction.

## Supporting information

S1 Fig Ovarian histology by H&E staining exhibited no difference between *Pik3ca^f/f^* and *Pik3ca^d/d^* mice. Ovarian histology of *Pik3ca*^*f/f*^ (a) and *Pik3ca*^*d/d*^ (b) mice.
(PDF)

S2 Fig The expression of phospho-AKT and total AKT in the uterus of *Pik3ca^f/f^* and *Pik3ca^d/d^* mice. Immunohistochemical staining of total phospho-AKT (a and b) and AKT (c and d) in *Pik3ca*^*f/f*^ (a and c) and *Pik3ca*^*d/d*^ (b and d) mice.
(PDF)
