# Supplementary material for: Pik3ca is required for mouse uterine gland development and pregnancy
Source: PLoS One. 2018 Jan 18;13(1):e0191433. doi: 10.1371/journal.pone.0191433 (PMC5773209; doi:10.1371/journal.pone.0191433)
Supplement: S1 Fig — Ovarian histology of Pik3caf/f (a) and Pik3cad/d (b) mice. (PDF) [file pone.0191433.s001.pdf]

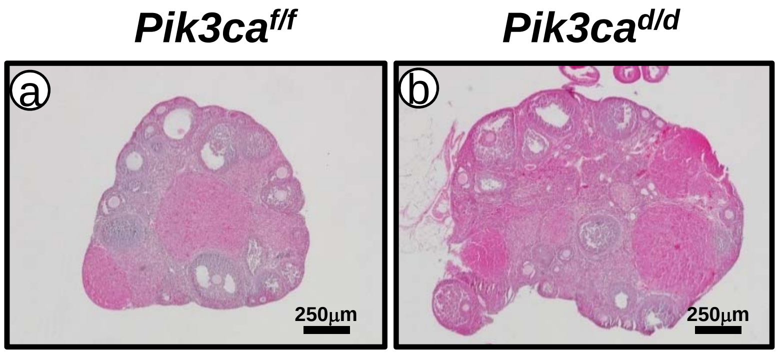

S1 Fig. Ovarian histology by H&E staining exhibited no difference between *Pik3ca*$^{f/f}$ and *Pik3ca*$^{d/d}$ mice.
