# Supplementary material for: Pik3ca is required for mouse uterine gland development and pregnancy
Source: PLoS One. 2018 Jan 18;13(1):e0191433. doi: 10.1371/journal.pone.0191433 (PMC5773209; doi:10.1371/journal.pone.0191433)
Supplement: S2 Fig — Immunohistochemical staining of total phospho-AKT (a and b) and AKT (c and d) in Pik3caf/f (a and c) and Pik3cad/d (b and d) mice. (PDF) [file pone.0191433.s002.pdf]

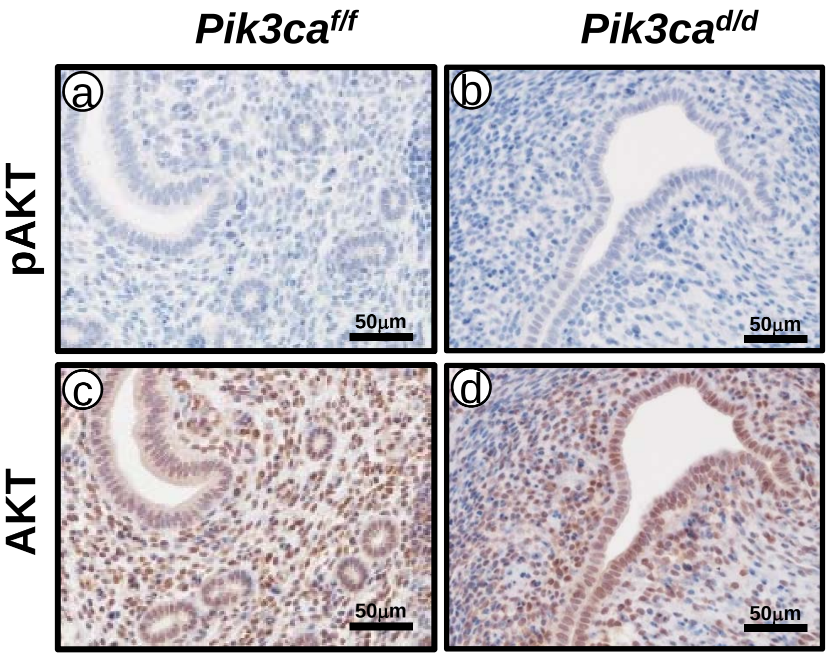

S2 Fig. The expression of total AKT and phosphor-AKT in the uterus of *Pik3ca*$^{f/f}$ and *Pik3ca*$^{d/d}$ mice.
